# Global differences in specific histone H3 methylation are associated with overweight and type 2 diabetes

**DOI:** 10.1186/1868-7083-5-15

**Published:** 2013-09-03

**Authors:** Åsa Jufvas, Simon Sjödin, Kim Lundqvist, Risul Amin, Alexander V Vener, Peter Strålfors

**Affiliations:** 1 Department of Clinical and Experimental Medicine, Linköping University, Linköping SE58185, Sweden

**Keywords:** Epigenetic, Histone methylation, Human, Obesity, Overweight, Primary mature adipocytes, Type 2 diabetes

## Abstract

**Background:**

Epidemiological evidence indicates yet unknown epigenetic mechanisms underlying a propensity for overweight and type 2 diabetes. We analyzed the extent of methylation at lysine 4 and lysine 9 of histone H3 in primary human adipocytes from 43 subjects using modification-specific antibodies.

**Results:**

The level of lysine 9 dimethylation was stable, while adipocytes from type 2 diabetic and non-diabetic overweight subjects exhibited about 40% lower levels of lysine 4 dimethylation compared with cells from normal-weight subjects. In contrast, trimethylation at lysine 4 was 40% higher in adipocytes from overweight diabetic subjects compared with normal-weight and overweight non-diabetic subjects. There was no association between level of modification and age of subjects.

**Conclusions:**

The findings define genome-wide molecular modifications of histones in adipocytes that are directly associated with overweight and diabetes, and thus suggest a molecular basis for existing epidemiological evidence of epigenetic inheritance.

## Background

The dynamics of chromatin regulate access to DNA and are therefore under tight control by the host cell and by external stimuli. Reversible covalent post-transcriptional modifications to histones are considered to form one of the major means by which gene transcription and DNA replication are controlled [1]. Histone modifications have been associated with transcriptional control since the discovery of histone acetylation [2]; hyperacetylated histones are linked to actively transcribed genes [2,3].

Methylation of histone H3 at lysine 4 is associated with sites of active gene transcription [4,5]. High levels of dimethylation and trimethylation (H3K4me2 and H3K4me3) are generally found near promoter regions of DNA. Trimethylation, particularly, is found at transcription start sites, while dimethylation flanks these sites of active genes [6,7]. Enhancers appear to host higher levels of monomethylated lysine 4. Dimethylation of lysine 9 (H3K9me2), on the other hand, is a modification found in heterochromatin throughout silenced genes [7] but is also found in actively transcribed genes [8]. Methylation of histones is a reversible and dynamic process that is catalyzed by specific and general histone methyltransferases and demethylases, which are, in turn, dependent on metabolic coenzymes and thus responsive to changes in energy supply and metabolic status [9].

Obesity and type 2 diabetes (T2D) are characterized by strong hereditary components in addition to such lifestyle-related factors as overeating and physical inactivity; however, no simple relation to gene variants has been discovered. Conversely, genome-wide association studies have uncovered a number of genes that are associated with increased risks of developing the conditions, but the identified genes are each associated with a very low risk and are widely distributed in the population as a whole [10-13].

It is clear that lifestyle and environmental exposure can cause long-lasting susceptibility or resistance to disease, even in later generations, suggesting non-genetic memory and inheritance. Epidemiological data and clinical and experimental studies indicate that nutritional conditions during early life can strongly influence later susceptibility to T2D. Epigenetic mechanisms have been used to explain the discovery that the famine experienced by pregnant mothers in the Netherlands in World War II affected the birth weights of their children, and their children’s later development of obesity and impaired glucose tolerance [14-16]. In addition, it was found that different starvation or surfeit experiences by parents and grandparents in Överkalix in northern Sweden during the late nineteenth and early twentieth centuries was associated with different susceptibilities to death from cardiovascular disease or T2D in their offspring [17]. Recently, a study of the whole population of Austria found a massively increased risk of diabetes in people born during or immediately after one of three different famines of the twentieth century [18]. In experimental animal studies, the importance of the intrauterine environment has been demonstrated [19-21], as well as a paternal non-genetic transgenerational inheritance of propensity for obesity and diabetes [22,23]. It has been suggested that methylation of DNA, modifications of histones, and noncoding RNA mediate epigenetic inheritance. Methylation of DNA and histone modifications have been shown to be affected by, for example, body mass index (BMI) [24], age [25], intrauterine environment [26-28], glucose exposure [29,30], and exercise [31].

In this study, we investigated whether there is a relation between overweight or obesity, T2D and genome-wide methylation of histone H3 at lysine 4 and at lysine 9 in isolated mature adipocytes.

## Results

We analyzed the global extent of H3K4me2, H3K4me3, and H3K9me2 in isolated primary mature adipocytes from subjects who were of normal weight, overweight, or overweight with type 2 diabetes. The extent of methylation was determined by SDS-PAGE and immunoblotting using site- and modification-specific antibodies. The extent of specific methylation was normalized for the total amount of histone H3 in each sample, and all values are the median value of three separate experiments. Hence, the extent of histone H3 methylation is determined as the fractional methylation of histone H3.

**Figure 1:**
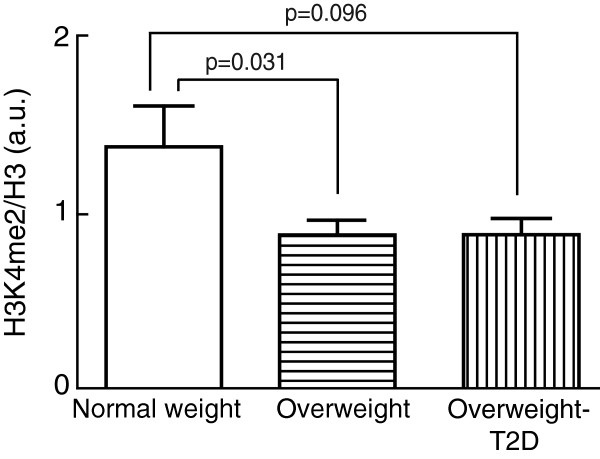
**Level of H3K4me2 in human adipocytes.** The level of H3K4me2 was determined in isolated adipocytes from normal-weight (BMI < 25 kg/m^2^), overweight (BMI ≥ 25 kg/m^2^), and overweight subjects with T2D. Bar graph shows the H3K4me2/total histone H3, mean ± SEM (*n* = number of subjects) in adipocytes from normal-weight subjects (*n* = 14), non-diabetic overweight subjects (*n* = 19), and overweight subjects with T2D (*n* = 10). Cells from all subjects were analyzed in three separate experiments and the median value was used in the subsequent analysis. a.u., arbitrary units; BMI, body mass index; SEM, standard error of the mean; T2D, type 2 diabetes.

The level of H3K4me2 was 37% lower in adipocytes from overweight subjects, whether non-diabetic or with T2D, compared with normal-weight non-diabetic subjects (Figure 1). Moreover, when combining the whole group of overweight (non-diabetic and T2D) subjects, the level of H3K4me2 was significantly lower (*P* = 0.009) than in the adipocytes from the normal-weight subjects (not illustrated).

**Figure 2:**
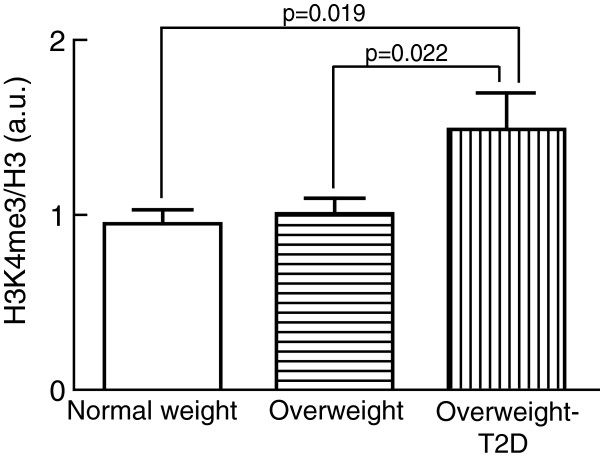
**Level of H3K4me3 in human adipocytes.** The level of H3K4me3 was determined in isolated adipocytes from normal-weight (BMI < 25 kg/m^2^), overweight (BMI ≥ 25 kg/m^2^), and overweight subjects with T2D. Bar graph shows the H3K4me3/total histone H3, mean ± SEM (*n* = number of subjects) in adipocytes from normal-weight subjects (*n* = 11), non-diabetic overweight subjects (*n* = 16), and overweight subjects with T2D (*n* = 10). Cells from all subjects were analyzed in three separate experiments and the median value was used in the subsequent analysis. a.u., arbitrary units; BMI, body mass index; SEM, standard error of the mean; T2D, type 2 diabetes.

In contrast, the level of H3K4me3 was 40% higher in adipocytes from overweight subjects with T2D than in normal-weight non-diabetic or overweight non-diabetic subjects (Figure 2).

**Figure 3:**
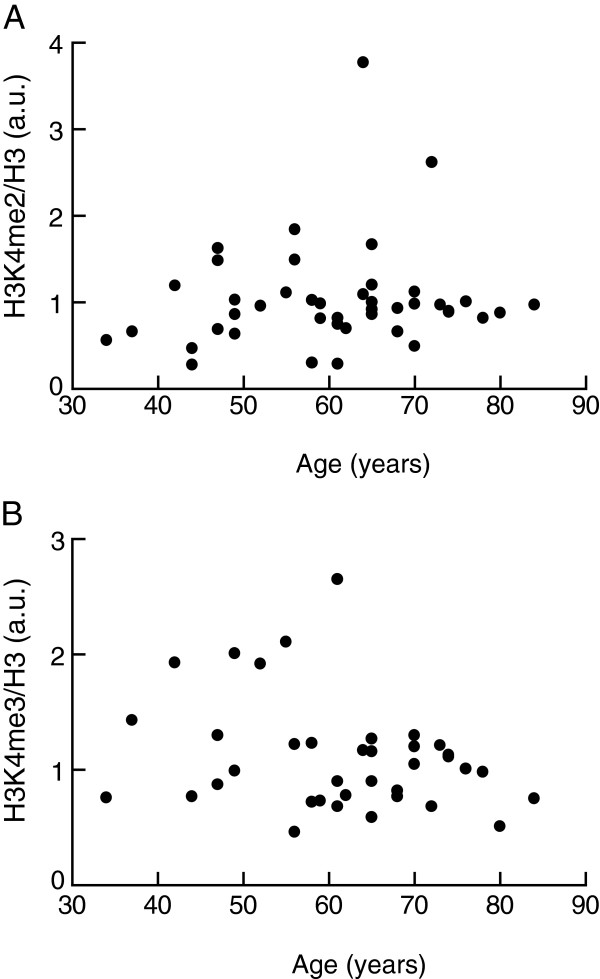
**Level of H3K4me2 and H3K4me3 in human adipocytes in relation to age of subjects.** The levels of H3K4me2 **(A)** and H3K4me3 **(B)** were determined in isolated adipocytes from normal-weight and overweight (BMI ≥ 25 kg/m^2^) subjects and overweight subjects with T2D, and plotted against the age of donor subjects. There was no significant correlation between level of modification and age: *P* = 0.39 **(A)**; *P* = 0.10 **(B)**. Cells from all subjects were analyzed in three separate experiments and the median value was used in the subsequent analysis. a.u., arbitrary units.

As an association between epigenetic changes and age can be expected and has indeed been observed [32], we examined whether there was any association between the extent of histone modification and the age of the subjects. However, we found no significant association between the global levels of H3K4 dimethylation or trimethylation in the isolated adipocytes and the age of the corresponding subjects (Figure 3).

**Figure 4:**
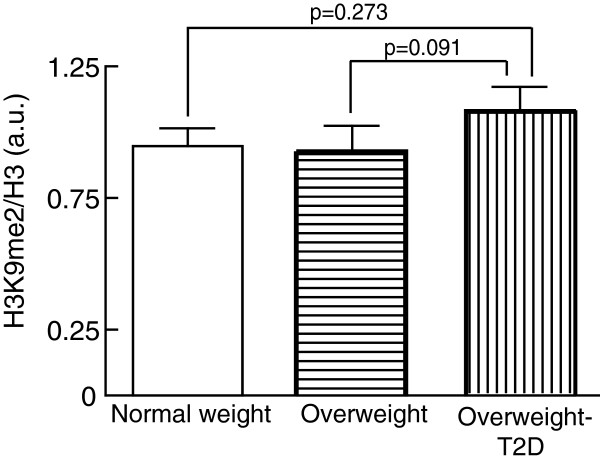
**Level of H3K9me2 in human adipocytes.** The level of H3K9me2 was determined in isolated adipocytes from normal-weight (BMI < 25 kg/m^2^, *n* = 7), non-diabetic overweight (BMI ≥ 25 kg/m^2^, *n* = 10), and overweight subjects with T2D (*n* = 10). Bar graphs show the H3K9me2/total histone H3, mean ± SEM (*n* = number of subjects). Cells from all subjects were analyzed in three separate experiments and the median value was used in the subsequent analysis. a.u., arbitrary units; BMI, body mass index; SEM, standard error of the mean; T2D, type 2 diabetes.

In contrast with H3K4-methylation, the level of H3K9me2 was similar in adipocytes from T2D and non-diabetic subjects and was not dependent on donor overweight (Figure 4).

## Discussion

Our findings reveal large genome-wide differences in the level of specific histone methylation in adipocytes from subjects with overweight or T2D compared with normal-weight and non-diabetic subjects. These differences were not related to the age of the subjects donating the adipocytes. The effects were restricted to H3K4 methylation, which is associated with actively transcribed genes, with no corresponding effects in the heterochromatin-defining H3K9 methylation. It is particularly interesting that overweight and T2D are associated with changes involving nearly half of the dimethylation and trimethylation levels at H3K4 in the adipocytes. This indicates that a large number of genes might be affected by the changed levels of modifications. The underlying cause of these differences probably originates from differences in activities of one or more of the involved methylases or demethylases, or their control. Histone methylation is a reversible process and we cannot exclude changes during surgical procedures and isolation or incubation of the cells, but our findings nevertheless demonstrate large genome-wide changes in overweight and T2D that are directly related to these specific histone modifications. Since most genetic variants associated with T2D appear to be linked to β-cell function and insulin release [10,11] our findings indicate a potential importance of the adipose tissue in hereditability of T2D. An epigenetic link to overweight and T2D is in line with the epidemiological studies discussed previously [14,15,17,18,26].

H3K4me2 is demethylated by LSD1, a FAD-dependent demethylase [33-35]. Interestingly, it has been shown that LSD1 has an increased expression in adipocytes from high-fat diet-fed mice and that adipose energy-expenditure genes are direct targets of repression by LSD1 [34]. Inhibition of LSD1 increases global H3K4 methylation in P19 embryonal carcinoma cells [36] and lowers the body weight of mice fed a high-fat diet [34]. Histone methyltransferase MLL3 catalyzes methylation of H3K4 [37]. Mice with mutations in the catalytic SET-domain of MLL3 show altered gene expression of a number of metabolic genes in adipose tissue, such as Rbp4 [38], which is associated with insulin resistance in human beings [39,40]. The mutant mice also exhibit an altered phenotype, with less adipose tissue and improved insulin sensitivity compared with control mice [38]. Collectively, these reports demonstrate that modifying the global levels of H3K4 methylation experimentally affects adiposity and sensitivity to insulin. This is further supported by experiments showing that the levels of H3K4me3 in PPARγ promoters correlate with expression levels of PPARγ during adipogenesis [41]. Interestingly, H3K9me2 was selectively enriched in the entire PPARγ locus in 3T3-L1 preadipocytes [42], and the level of H3K9me2 correlated inversely with induction of PPARγ in both murine and human adipogenesis [42]. However, globally we found no correspondence between levels of H3K9 and H3K4 methylation in the mature adipocytes of normal-weight, overweight, or diabetic individuals.

It may be that histone modifications do not determine sites of active transcription, but rather reinforce the effects of nucleosome binding during transcription, for example, in response to the targeting actions of noncoding RNAs [1]. As such, our findings are indicators of large genome-wide changes in transcriptional activities associated with overweight and diabetes, which may be involved in an epigenetically affected propensity for these common disorders. In the future, it will be interesting to analyze to what extent particular sets of genes are affected in different individuals, who may be of normal weight, overweight, or diabetic.

## Conclusions

Our findings define extensive genome-wide molecular modifications of histones in adipocytes that are directly associated with overweight and diabetes. Effects were restricted to H3K4 methylation, which is associated with actively transcribed genes, with no corresponding effects in the heterochromatin-defining H3K9 methylation. Changes involved 30% to 40% of the dimethylation and trimethylation levels at H3K4 in the adipocytes, indicating that a large number of genes might be affected by the changed levels of modifications. The findings suggest a molecular basis for existing epidemiological evidence of epigenetic inheritance.

## Methods

### Subjects

The study was approved by the Regional Ethics Board at Linköping University and has been carried out in accordance with the declaration of Helsinki; all patients obtained written information and gave their informed approval before surgery. Subcutaneous abdominal fat tissue was obtained during elective surgery on patients at the University Hospital, Linköping and Norrköping. Clinical data are summarized in Table 1.

**Table 1:** Characteristics of participating subjects

	**Normal weight**	**Overweight**	**T2D**
	**(BMI < 25 kg/m**^**2**^**)**	**(BMI > 25 kg/m**^**2**^**)**	
Female/male	14/0	19/0	8/2
Age (years)	64.4 ± 8.7	60.2 ± 11.4	55.2 ± 15.2
BMI (kg/m^2^)	22.4 ± 1.5	34.5 ± 8.3	41.4 ± 10.8
Fasting glucose (mmol/l)	5.8 ± 1.0	6.2 ± 8.9	8.0 ± 0.5
Fasting insulin (pmol/l)	73.0 ± 64.0	54.5 ± 34.4	112.0 ± 114.2

Mean ± SD.

### Isolation and incubation of adipocytes

Adipocytes were isolated from adipose tissue samples by collagenase digestion (type 1, Worthington, NJ, USA) in modified Krebs-Ringer solution [43]. Following overnight incubation [44], cells were washed with the modified Krebs-Ringer solution and incubated with 0.1 μM N6-phenylisopropyl adenosine and 2.5 μg/ml adenosine deaminase for 10 min, to control the intracellular concentration of cyclic AMP and establish a standardized level of basal lipolysis [45]. Cells were separated from the medium by centrifugation through dinonyl phthalate and were then immediately dissolved in SDS and β-mercaptoethanol with protease and phosphatase inhibitors, frozen within 10 seconds and thawed in boiling water for further analysis [43].

### SDS-PAGE and immunoblotting

Proteins were separated by SDS-PAGE (14.5% acrylamide) [46] and transferred to a polyvinylidene difluoride blotting membrane (Immobilon-P, Millipore, MA, USA). The extent of H3K4 and H3K9 methylation was analyzed with antibodies against H3K4me2, H3K4me3, or H3K9me2 (Active Motif, Carlsbad, CA, USA). These antibodies are specific for dimethylation or trimethylation, such that the H3K4me2-specific antibodies do not cross-react with H3K4me3. Membranes were stripped of bound antibodies (2% SDS, 62.5 mM Tris, 100 mM β-mercaptoethanol, 60°C, 30 min) and the amount of histone H3 was determined in each sample with antibodies against histone H3 C-terminus (Active Motif), to calculate the ratio of histone H3 methylation to the amount of histone H3. To allow comparison between different gels, a standard sample (a mixture of aliquots from 23 subjects) was run in duplicate on every gel and all samples were normalized against the mean of the standard sample. Antibodies were detected using horseradish peroxidase conjugated IgG secondary antibody (Santa Cruz Biotechnical, Santa Cruz, CA, USA) and ECL-plus (Amersham Biosciences, Little Chalfont, Bucks, UK) using chemiluminescence imaging (LAS 1000; Image Gauge v.3.0, Fuji, Tokyo, Japan). Linearity of the antibodies’ responses was ascertained (Additional file 1: Figure S1) and the amounts of each sample subjected to SDS-PAGE were adjusted to fall within this linear range. For the calculations, the median of three separate immunoblottings was used for each of the 43 subjects. Groups were compared with two-tailed Student’s *t* test, using GraphPad Prism v.5.00 (GraphPad software Inc., San Diego, CA, USA).

## Abbreviations

a.u.: Arbitrary units; BMI: Body mass index; H3K4me2: Histone H3 dimethylated at lysine 4; H3K4me3: Histone H3 trimethylated at lysine 4; H3K9me2: Histone H3 dimethylated at lysine 9; IgG: Immunoglobulin G; SEM: Standard error of the mean; T2D: Type 2 diabetes; SD: Stadard deviation.

## Competing interests

The authors declare that they have no competing interests.

## Authors’ contributions

ÅJ, AVV, and PS conceived and designed the experiments. ÅJ, SS, KL, and RA performed the experiments. ÅJ, SS, KL, AVV, and PS analyzed the data. ÅJ, AVV, and PS wrote the paper. All authors read and approved the final manuscript.

## Supplementary Material

Additional file 1: Figure S1 Linearity of immunoblotting with antibodies against H3K4me2, H3K4me3, H3K9me2, and H3 C-terminus. Click here for file
