# Supplementary material for: Global differences in specific histone H3 methylation are associated with overweight and type 2 diabetes
Source: Clin Epigenetics. 2013 Sep 3;5(1):15. doi: 10.1186/1868-7083-5-15 (PMC3766271; doi:10.1186/1868-7083-5-15)
Supplement: Additional file 1: Figure S1 — Linearity of immunoblotting with antibodies against H3K4me2, H3K4me3, H3K9me2, and H3 C-terminus. [file 1868-7083-5-15-S1.pdf]

Figure S1

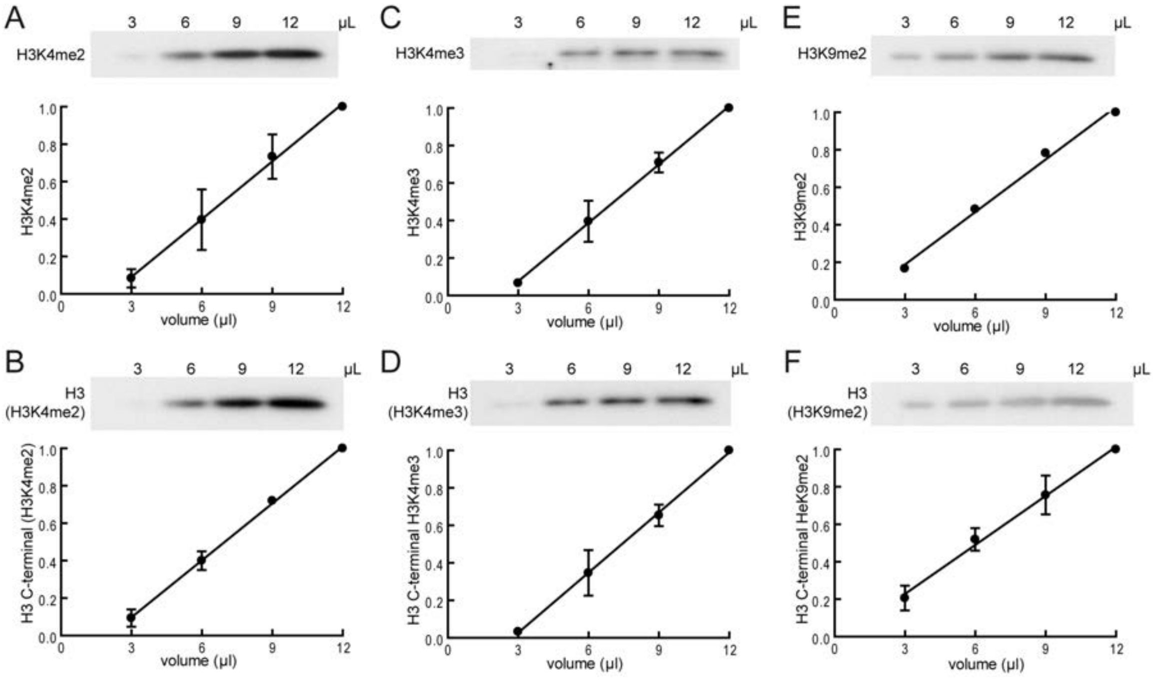

Figure S1 Linearity of immunoblotting with antibodies against H3K4me2, H3K4me3, H3K9me2 and H3 C-terminus

A mix composed of samples from 23 subjects was analyzed by SDS-PAGE and immunoblotting. 3, 6, 9 or 12 µL mix, as indicated, was subjected to analysis with each one of the modification specific antibodies followed by stripping and re-blotting with antibody against the H3 C-terminal. Intensities were normalized against the intensity of the 12-µL sample. (A) H3K4me2. (B) H3 C-terminal after removing the H3K4me2-specific antibody. (C) H3K4me3. (D) H3 C-terminal after removing the H3K4me3-specific antibody. (E) H3K9me2. (F) H3 C-terminal after removing the H3K9me2-specific antibody. The mean ±SEM of three experiments is shown.
